# Outdoor activities foster local plant knowledge in Karelia, NE Europe

**DOI:** 10.1038/s41598-023-35918-7

**Published:** 2023-05-27

**Authors:** G. Mattalia, I. Svanberg, S. Ståhlberg, N. Kuznetsova, B. Prūse, V. Kolosova, M. A. Aziz, R. Kalle, R. Sõukand

**Affiliations:** 1 Department of Environmental Sciences, Informatics and Statistics, Università Ca’ Foscari Venezia, Venice, Italy; 2 Institute for Russian and Eurasian Studies, Uppsala Universitet, Uppsala, Sweden; 3 Università Cattolica del Sacro Cuore, Milan, Italy; 4 Vrije Universiteit, Amsterdam, The Netherlands; 5 Estonian Literary Museum, Tartu, Estonia

**Keywords:** Environmental social sciences, Socioeconomic scenarios

## Abstract

Wild edible plants, particularly berries, are relevant nutritional elements in the Nordic countries. In contrast to decreasing global trends, approximately 60% of the Finnish population is actively involved in (berry) foraging. We conducted 67 interviews with Finns and Karelians living in Finnish Karelia to: (a) detect the use of wild edible plants, (b) compare those results with the published data about neighbouring Russian Karelians, and (c) document the sources of local plant knowledge. The results revealed three main findings. First, we observed a similarity in wild food plant knowledge among Karelians and Finns from Karelia. Second, we detected divergences in wild food plant knowledge among Karelians living on both sides of the Finnish–Russian border. Third, the sources of local plant knowledge include vertical transmission, acquisition through literary sources, acquisition from “green” nature shops promoting healthy lifestyles, childhood foraging activities performed during the famine period following WWII, and outdoor recreational activities. We argue that the last two types of activities in particular may have influenced knowledge and connectedness with the surrounding environment and its resources at a stage of life that is crucial for shaping adult environmental behaviours. Future research should address the role of outdoor activities in maintaining (and possibly enhancing) local ecological knowledge in the Nordic countries.

## Introduction

Wild edible plants are important nutritional elements in circumpolar countries^1–3^. Among these plants, berries are the most popular wild foods, often harvested in the summertime and then processed for consumption throughout the long hard winter^4, 5^.

Historically, berry picking was a common activity in the Nordic countries for both household and recreational purposes^6–8^. Contrary to the global trend, in Finland berry harvesting has been quite stable over the last few decades, with approximately 60% of the Finnish population actively involved in this activity^7^. In remote regions, this activity was also an important source of income, following the principle of *jokamiehenoikeus* (lit: everyman’s right) which allows berry and mushroom picking on public and private land for both personal and commercial purposes^9^. Currently, only a small quantity of berries naturally produced in forests are actually harvested^7, 10^. The few local companies involved in the berry industry no longer rely on local pickers, and in the Nordic countries most berries used in the jam industry are harvested by seasonal workers mainly from Thailand^11^. Over the past century, the relationship between Nordic people and berries, and more generally wild food plants, has evolved. Especially in the past few decades, the concept of the “New Nordic Cuisine”, based on the valorisation of local food resources and local terroirs, has turned a subsistence activity into high-end gastronomy^8, 12^.

Despite the importance of wild food plants and the associated knowledge (“LEK or local ecological knowledge”^13^), the Nordic countries have been little studied from an ethnobotanical perspective. A few exceptions apply to the historical aspect of local ecological knowledge related to wild edible plants which were investigated extensively by Svanberg and colleagues, e.g. in the Faroe Islands^14^, Sweden^15^, and Iceland^16^, and also by Alm and colleagues^17, 18^ and some other authors^19, 20^. Apart from a few publications focused on economic and social trends involving berries^21–23^, the only Finnish ethnobotanical study was conducted in Sápmi (the northernmost area of Fennoscandia, also known as Lapland) among the Skolt Sámi^24^.

To fill this knowledge gap, we conducted extensive fieldwork in Finnish Karelia to identify trajectories in the use of wild food plants, within the framework of the ERC project “Divided Generations” aimed at documenting LEK related to wild food plants and understanding its diachronic evolution in different political contexts of Northern and Eastern Europe^25–28^.

After the independence of the Grand Duchy of Finland from the Russian Empire in 1917, the province of Karelia remained part of Finland. During WWII, however, the eastern part of Karelia was first occupied and then annexed by the Soviet Union. This resulted in the evacuation of a large portion of the Karelian and Finnish population to Finland. Since then, Finnish-speakers from Karelia and their descendants, as well as Karelians who speak the currently endangered Karelian language, have lived on both sides of the Finnish–Russian border. This allows for cross-cultural comparison between Finns and Karelians living in the eastern Finnish province of North Karelia, and a cross-border comparison between Karelian-speakers living on either side of the Finnish–Russian border.

Within this framework, we aimed to:

- Document current and past uses of wild food plants in North Karelia (Finland) by Karelians evacuated to this territory in 1940 and by the local Finnish population of the same age, as well as by the descendants (children and grandchildren) of both groups;
- Detect similarities and differences in the use of wild food plants among Karelians and Russians living in the Republic of Karelia, Russian Federation;
- Identify factors which might have influenced local plant knowledge transmission in Finnish Karelia.

## Results

### The use of wild food plants in Finnish Karelia

We documented the current and past use of 47 wild food plant taxa, belonging to 21 families, among Finns and Karelians of Finnish Karelia. Seven taxa were used only by Karelian interviewees, 15 only by Finns, and 25 were shared between the two groups (see table in the Appendix). The Jaccard Similarity Index (JI) was 59% when all current plant taxa were included and 73% when only current plants mentioned by at least three informants were considered. *Carum carvi* was mentioned by four Finnish interviewees but by no Karelians.

The four most cited wild food plants among Finns and Karelians were shared between the two groups (Table 1). The most common families were Ericaceae (320 DUR among Finns, 354 DUR among Karelians) and Rosaceae (215 DUR among Finns, 264 DUR among Karelians).

**Table 1:** Top-5 wild food plants among Finns and Karelians in Finnish Karelia, DUR = detailed use reports.

Finns (total number of DUR = 662)	Karelians (total number of DUR = 716)
*Vaccinium myrtillus* L. (144 DUR Finns; 153 DUR Karelians)	
*Vaccinium vitis-idaea* L. (137 DUR Finns; 152 DUR Karelians)	
*Rubus idaeus* L. (74 DUR)	*Rubus chamaemorus* L. (89 DUR)
*Rubus chamaemorus* L. (72 DUR)	*Rubus idaeus* L*.* (81 DUR)
*Fragaria vesca* L. (41 DUR)	*Vaccinium oxycoccus* L. (40 DUR)

Among those who mentioned wild food plant use in either group, the average number of used plants per person was around nine, yet the range was higher among Finns (from 2 to 27 taxa) than Karelians (from 5 to 18 taxa). On average Finns recalled 20 wild food plant uses (DUR) per interviewee (ranging from 4 to 38), while this number was slightly higher among Karelians at 22 DUR per interviewee (ranging from 5 to 41). However, in both groups, most of the uses referred to the past (over 50%), with approximately one-third referring to continuous uses (i.e. passed down from previous generations) and around 10% representing recently adopted uses. Temporary uses (those adopted and abandoned in a short period of time) represented only 2% of the DUR in both groups. The most common food uses were snacks (120 DUR among Finns, 114 DUR among Karelians), followed by jam and pies among Finns (78 and 66 DUR, respectively), and pies and porridge among Karelians (83 and 69 DUR, respectively).

Among the plants mentioned in Finland, for which parts other than their fruits were used, the following are notable: the sap of *Betula* sp. as a drink (9 DUR among both Karelians and Finns), the aerial parts of Urtica dioica mainly for soup or tea (15 DUR among Karelians and 24 DUR among Finns), and the leaves of *Rumex acetosa* mainly as a snack in childhood (13 DUR among Karelians and 16 DUR among Finns). Among Finns, fresh shoots of *Picea abies* and the leaves of *Oxalis acetosella* as snacks were also found to be relevant (13 and 12 DUR, respectively; but only 9 and 5 DUR, respectively, among Karelians).

**Figure 1:**
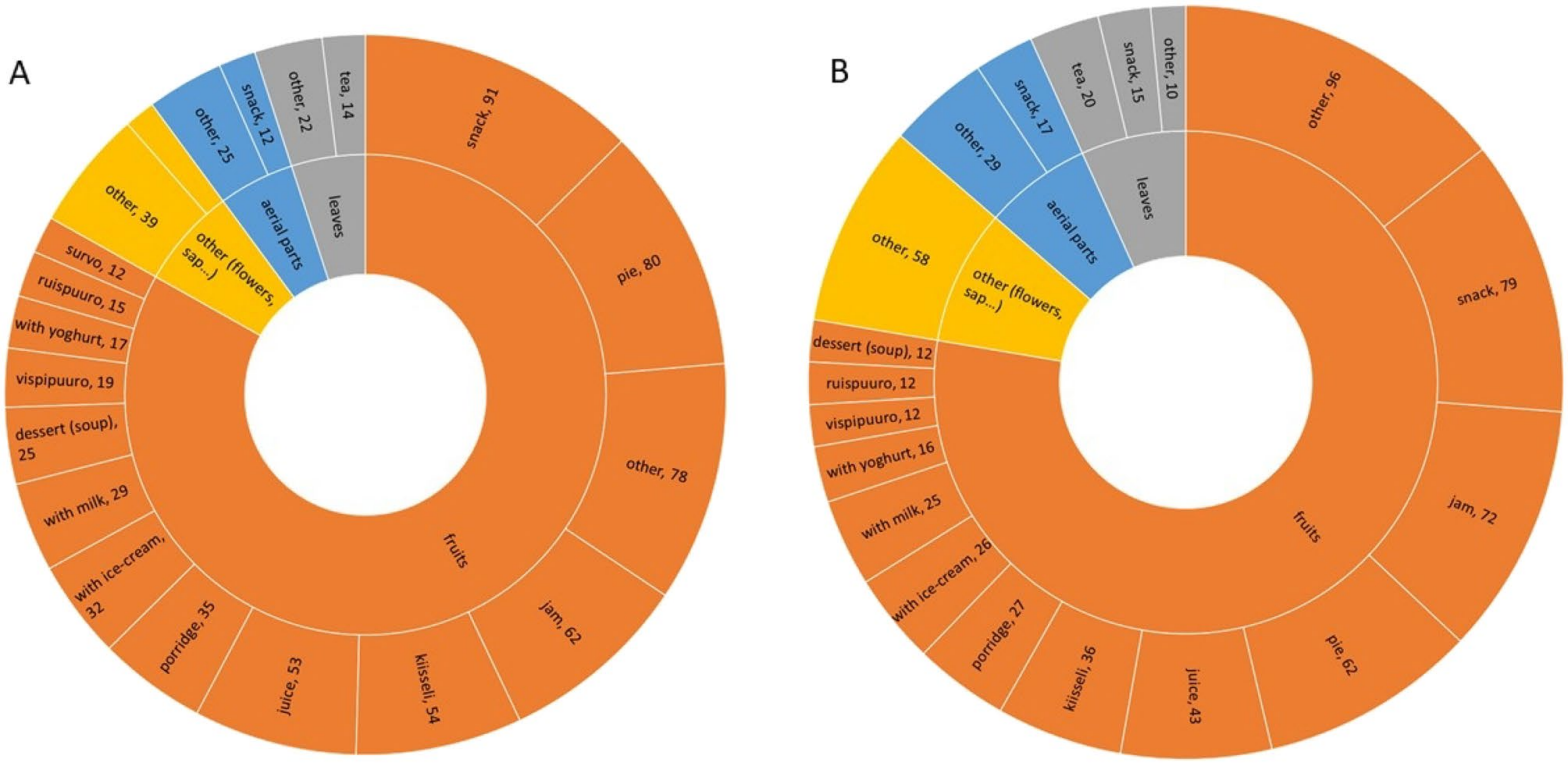
Graphical representation of detailed use reports and plant parts used among Karelians (**a**) and Finns (**b**) living in North Karelia, Finland. Number of DUR respectively: 724 and 667, all DUR below 10 are included under the category “other”. Legend: *ruispuuro*: rye porridge with lingonberry mash; *vispipuuro*: whipped semolina porridge with lingonberry juice; *kiisseli*: a viscous berry soup thickened with potato starch, which can be eaten hot or cold; *survo*: mashed berries.

Berries were the most common wild food plants used. In fact, fruits constituted over 80% of the DUR (n = 1120, 518 DUR among Finns, 602 DUR among Finnish Karelians) among the plant parts used. Berries were also the most common wild plant-based ingredient in food preparations (Fig. 1).

After snacks and the production of jam, the most frequently mentioned preparations were pies, porridge, cordial and *kiisseli* or kissel (thick fruit soup) (Fig. 1), followed by berry soups and *survo* (mashed berries).

Pastries are an important part of Karelian and Finnish culture. Our interviewees reported *Vaccinium myrtillus* and *V. vitis-idaea*, and also *Rubus chamaemorus* and *R. idaeus*, as the most used fruits for large open pies among both Finns and Karelians.

Porridge included two traditional types: *ruispuuro* (rye porridge with lingonberry mash) and *vispipuuro* (whipped semolina porridge with lingonberry juice). Moreover, interviewees also frequently put whole berries into porridge, now more often than before, as they also use frozen berries. According to our interviewees, the most common berries for porridge were *Vaccinium vitis-idaea* (*ruispuolukkapuuro*) and to a lesser extent* V*. *myrtillus*, while up to seven other fruits (including *Sorbus aucuparia* and *Rosa* spp.) are also added to porridge.

Kissel (*Kiisseli* in Finnish) is another berry-based product common to other Nordic countries. It consists of a viscous soup made from berries and potato starch, which can be eaten hot or cold. For kissel, the genus *Vaccinium*, including *V. myrtillus*, *V. oxycoccus* and *V. vitis-idaea*, was the most commonly used, followed by *Rubus* spp. (mainly *R. arcticus*, *R. chamaemorus* and *R. idaeus*).

### Comparative use of wild food plants among Karelians in Finland and Russia

We documented the use of 47 species common to Karelians living in Finland and Russia. Additionally, six species were mentioned only in Finland and 19 only in Russia. The Jaccard Similarity Index was 47% when all currently used plant taxa were included and 67% when only the currently used plants mentioned by at least three informants were considered. *Picea abies* was mentioned by seven Finnish Karelian interviewees but by no Russian Karelians. Five taxa were mentioned only in Russian Karelia: *Empetrum nigrum* as a snack or drink (seven interviewees), *Prunus padus* as a snack (nine interviewees), and *Rubus nessensis* also as a snack (five interviewees). *Hypericum maculatum* and *Populus tremula* were both mentioned by four Karelians in Russia for making tea and as fuel for smoking fish, respectively. It is worth mentioning that *Rubus nessensis* is not present in Finland^29^.

**Figure 2:**
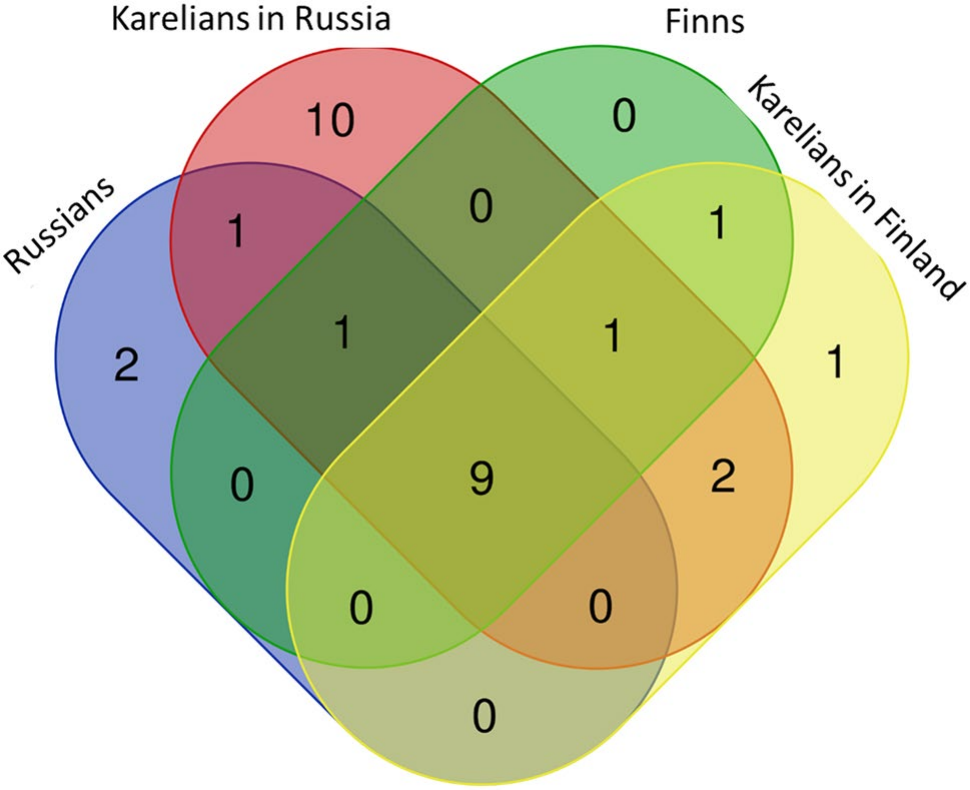
Number of shared species among Karelians living across the Finnish–Russian border, Russians living in the Republic of Karelia and Finns living in North Karelia.

Karelians living in Finland and Russia shared the most widely used wild food plants (all berries), although in a slightly different order of frequency (Fig. 2).

**Figure 3:**
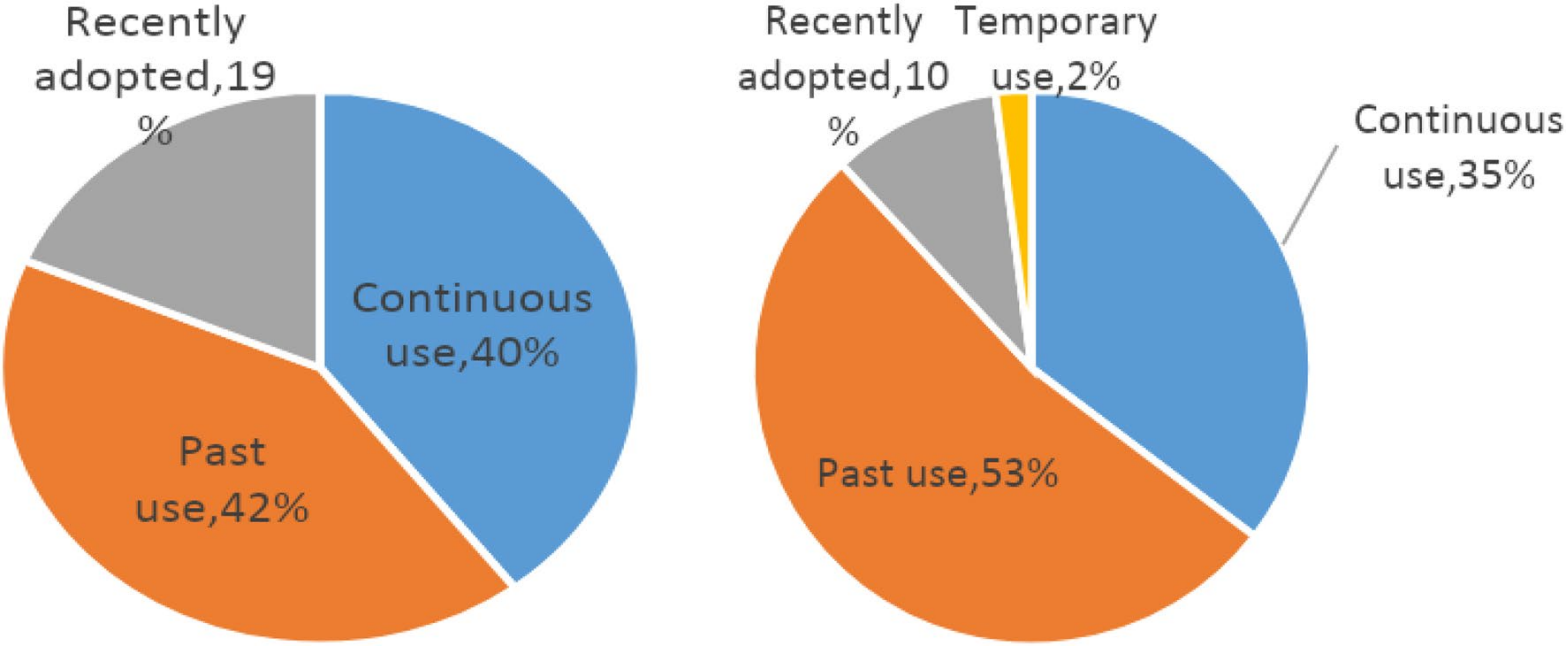
Comparison of the periods of use of wild food plants among Karelians living in Russia (left) and in Finland (right).

Compared with Karelians living in Finland, those living in Russia reported 11% more past uses, a slightly higher number of continuous uses, and 9% more recently adopted uses (Fig. 3).

**Figure 4:**
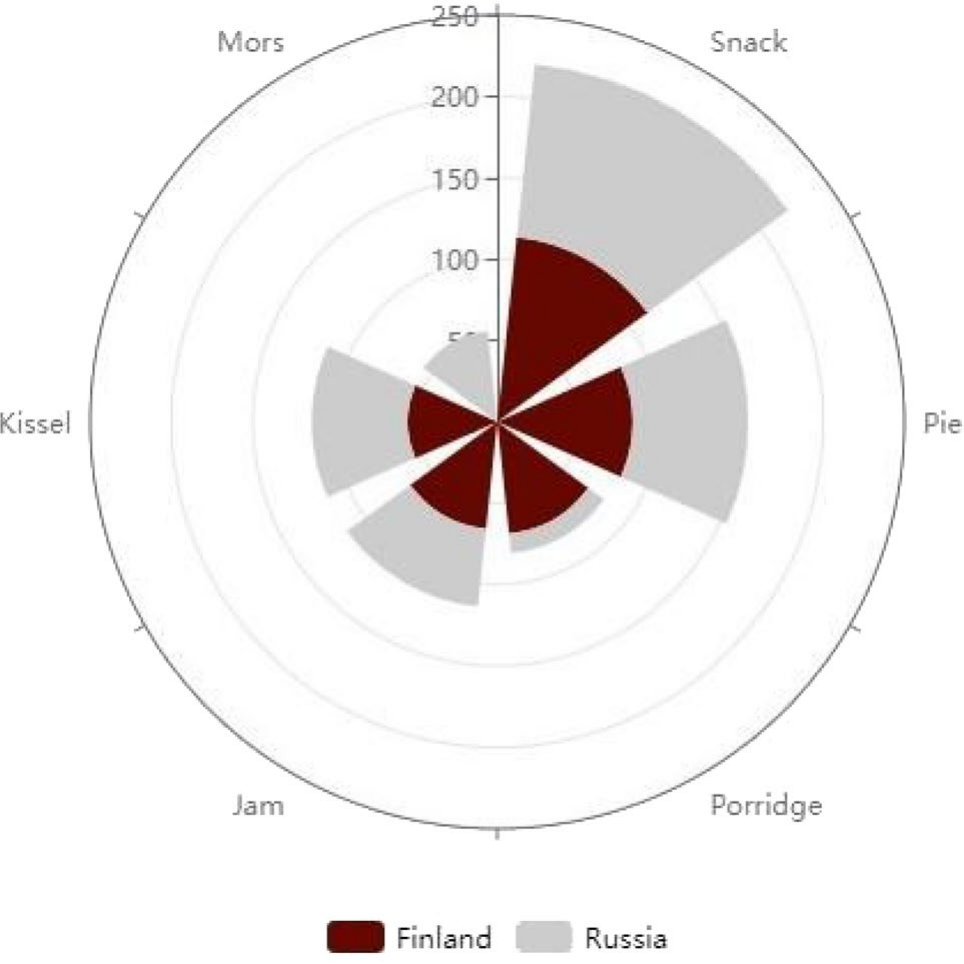
Top-5 most common food preparations among Karelians living in Finland and Russia, according to the number of DUR.

Among both groups, the most common food uses were snacks, pies, jam, and kissel, while porridge was reported only in Finland and *mors* reported only in Russia (Fig. 4). *Mors* is a drink made by adding sweetened boiled berry pulp to hot water.

### Local plant knowledge transmission in Finnish Karelia

#### Interviewees reported five sources of knowledge

First, vertical knowledge transmission was mentioned (e.g. knowledge from older relatives, e.g. “mother taught me how wild plants can be used in food”). Second, written resources were mentioned, including both online (e.g. “nowadays we learn everything from there [the Internet]”) and offline resources (e.g. “I read it in cookbooks” and “[the recipe] was in a magazine”). As stated by a Finnish interviewee (woman born in 1939), “I read *Mihin marjamme kelpaavat* [‘What our Berries are Useful for’, a book by Toivo Rautavaara (1905–1987), a famous post-war promoter of edible wild plants in Finland]. It had all these [plants]. […] Then there's a thick book: *Luonto, paras lääkitsijä* [‘Nature, the best Healer’, a book byAlfred Vogel (1902–1996)] […] It contains pretty good advice”.

Third, interviewees mentioned two activities related to school which involved wild plants. A vast majority of the interviewees recalled a mandatory herbarium activity that lasted until the end of the 1960s, in which up to 120 specimens had to be collected and their scientific names learned verbatim. Some other interviewees claimed that this activity has been partially resumed since the 1990s, and, starting in the 2000s, children have had to take photos of certain wild plants instead of collecting them. The second activity was recalled by a few elderly interviewees. In the post-war years, when food was scarce, and until the 1960s, there was a mandatory activity to collect lingonberries during a school trip in autumn. Alternatively, children collected them with parents and then brought the berries to school. A certain minimum quantity from each child was required (from one to five L were reported, depending on the age of children), and lingonberries were used in the school canteen (e.g. to prepare berry soups and kissel). This was part of providing food supplies to school, as there were no official school meals provided by the state at that time. Also, parents brought food to school according to their ability and means (e.g. rye, blueberries, strawberries, and potatoes were mentioned). However, these kinds of school activities happened only once a year, and children were involved in various other foraging activities with their families throughout the year, as foraging was a means of survival and enriching the diet in the lean post-war years.

The fourth knowledge source is outdoor activities, which are based on the long-term experiential relationship with non-human nature, including among other things, fishing, camping, skiing, berry and mushroom picking, canoeing, and walking in the forest^30–32^. In the Nordic countries, outdoor activities are performed from a very young age (kindergartens include several hours of playing outside regardless of weather conditions)^33^. These activities are pursued by both urban and rural inhabitants, either in rural or peri-urban contexts, during leisure time, including weekends and summer holidays^34^.

Partially in connection with this fourth point, there is now a fifth manner of knowledge transmission: “specialist” knowledge is transferred from health food store owners to customers. For instance, a Karelian woman (born in 1983), who visits an organic food shop in Nurmes (near Lieksa, in the study area), said, “[there are] flamonoids [flavonoids], fibres and vitamins [in berries and mushrooms]. That’ s a lot, just think of it, as they are taken from nature, [especially] now when those superfoods have become available. Berries are imported from abroad, for example the goji-berry. These [imported berries] contain lots [of flavonoids, fibres and vitamins] and they are advertised as so healthy. But if you think about how many berries there are and how much clean nature there is in Finland. […] These [local berries] are advertised mainly in those health food stores.” Another interviewee (Finnish woman born in 1958) confirmed, “in Finland, we have a huge amount of our own superfoods”. Likewise, a Karelian woman (born in 1960) mentioned, “I get nettle seeds from my neighbour’ s hill. They are really a superfood”.

A shift of perception among the importance of different approaches to the relation to non-human nature with regard to knowledge sources was detected. An interviewee (Finnish man born in 1941) pointed out that “[people] were closer to nature [in the past]. Now everything has to be proved through research”. A different way of conceiving connections of people with nature was expressed by a Finnish woman (born in 1952), “I am more interested in [pure] knowledge than in exploiting nature. I want to know, not use. I feel happy when I see berries, mushrooms and plants. I have learned about them from books. I have something of a judicial relationship, I am not a ‘nature person’, although nature is something very close to me.”

Finally, some narratives expressed how younger generations have a different/lower contact with non-human nature: “Millions of litres of both berries and mushrooms remain unpicked in the forest. I think the problem is that my generation still knows something and also how to move about in the forest. But my sons’ generation is too urbanised, their outdoor playing was limited, and they lack the skills. In Helsinki, there are children who have never seen a live cow. [There is] no contact with the countryside or forest ‒ you can buy everything from the shop. We used to go with our children to the forest, they know how to move about, but lack the desire to do it. They don’t gather anything” (Karelian man, born in 1965). Another interviewee (Karelian man, born in 1965) shared his childhood memories about his family’s summer activities: “We gathered wild strawberries [*Fragaria vesca*] in Suhmura and at our summer cottage in Nurmijärvi. As a child, you needed to get just a small bowl of berries and it was a great treat. We ate the berries with milk and sugar. Still a wonderful taste memory. […] [Now] my boys don’t [collect anything]. They are city fools.”

## Discussion

Our results yielded three main findings. First, we observed a similarity in wild food plant knowledge among Karelians and Finns in North Karelia, Finland. Second, we detected divergences in wild food plant knowledge among Karelians living on both sides of the Finnish–Russian border. Third, we identified  five main mechanisms by which North Karelian inhabitants acquire knowledge about wild food plants.

### Blended ethnobotanical knowledge in Finland, but divergent knowledge among Karelians across the Finnish–Russian border

The first main finding of the study concerns the similarity in wild food plant knowledge among Karelians and Finns in Karelia, which may be due to the fact that the studied groups themselves, both in Finland and Russia, are mixed. Between 1940 and 1944, a mass forced displacement took place from the Karelian Isthmus, Border Karelia, and Ladoga Karelia to Finland^35^. Over 400,000 people, mostly farming households, were evacuated from the border area before the advancing Soviet army^35, 36^. Since then, Finns from other areas have also migrated to Finnish Karelia, and local Finns sometimes go to study or work elsewhere in the country and upon returning home bring with them new ethnobotanical knowledge. We argue that this displacement and mobility has likely facilitated the incorporation of Finnish elements into Karelian LEK, thus leading to its creolisation. Along this line is the “Finnicisation” of Karelian pastry recipes, and in particular *karjalanpiirakka*, a rice-, potato-, or carrot-filled pie, traditionally an identity marker of Karelians, which has now become a national Finnish dish available in any supermarket in Finland^30, 37^. We also acknowledge the common historical roots of Karelians and Finns and possible long-term similarities in their use of wild food plants.

The second main result involves the divergence in wild food plant knowledge between Karelians living in Finland and those in Russia. This dissimilarity may be due to the long period of time (over 70 years) in which the two groups have lived in different political, social, and economic contexts, and under different culinary influences (availability of ingredients in shops, cookbooks, food fashions, TV shows, etc.), resulting in the two groups gradually being assimilated into Finnish and Russian culture, respectively. Despite a common history and shared cultural identity in the past, in Finland, Karelian LEK appears to be creolised with Finnish LEK. Similarly, Karelians in the Russian Federation have more in common with local Russians than with the Karelians across the Finnish border. We acknowledge that some differences may also be due to slightly different classifications. Specifically, there is a partial overlap of the concept of Russian *mors* and Finnish “juice” (*mehu*), as Russian *mors* might at least partially intersect with berry drinks reported under “juice” in Finnish Karelia, especially among the oldest generation. Before the 1950–1960s, to make *mehu*, both Finns and Karelians simply boiled berries in water, filtered the liquid part, and then preserved it in bottles, sometimes adding sugar. Starting from the 1950s, an originally German device called a “steam juicer” (*mehumaija*) started penetrating the Finnish mass market, and both Finnish and Karelian interviewees switched to using it for making *mehu*. Steam juicers also existed in the USSR starting in the 1960–1970s, but Russian Karelians apparently did not use them. A partial overlap also occurs within the concepts of porridge in Finnish Karelia and *mousse* in Russian Karelia. Nevertheless, this divergence in knowledge appears mostly to be due to the past decades of separation, accompanied by the downplay and destruction of Karelian cultural values and traditional practices in the Soviet Union and Russia. In Finland, the policy towards Karelians has been different as Karelia and the epic *Kalevala* are considered part of Finnish national heritage and have been appropriated by mainstream society.

### Mechanisms of wild plant knowledge acquisition among Karelians and Finns in North Karelia

The third main finding of the study involves the identification of five mechanisms of wild food plant knowledge acquisition. Two of these, namely vertical knowledge transmission and literary sources, are fairly well-known mechanisms^38^. The other three mechanisms deserve closer attention.

First, childhood activities performed at school, especially after WWII when Finland suffered from a scarcity of food, gave rise to two perceptions of wild food plants among elderly individuals. On the one hand, people had to learn more about wild nature because there was no importation of goods to North Karelia. On the other hand, after the war people refused wild foods, partly because people had extensively substituted traditional foods for several years. The collection of wild berries was crucial for the older generations, who had survived the first decades after Independence (starting in 1917), Civil War (1918), and WWII (1939–1944) when Finland was attacked by the Soviet Union. Finland lost territories, had to resettle the population from Karelia, and pay a heavy war indemnity to the Soviet Union, which put a strain on the national economy ^1^. To face those difficult times, until the 1960s, the Finnish government supported the collection of berries by granting special school holidays to allow pupils to devote time to berry picking and other agricultural activities with their families^3^. Visuri^39^ also noted that starting in 1948, schools had to provide free school meals. However, in several regions, children were still expected to contribute by picking berries for the school canteen. This activity could potentially provide additional support to the instilling of foraging habits in children, although their parents and grandparents also picked berries and thus they also received family models from an early age. The second, context- specific mechanism is related to the Scandinavian concept of *friluftsliv*^40^, or open-air lifestyle, that today is a “cultural marker” of the Nordic countries, which was further fostered by the recent COVID-19 pandemic. We argue that these activities may have influenced knowledge about and connectedness with the surrounding environment^32^ and its resources at a stage of life that is crucial for shaping adult environmental behaviours^41, 42^. The third mechanism, which possibly applies only to part of the (adult) population, involves knowledge acquisition from “green” natural/health product shops, which promote healthy lifestyles^42^.

Our study suggests a “connection due to need” trajectory in the attitudes towards wild food plants in Finnish North Karelia. This trend, partially detected by Saastamoinen et al.^21^, has included a partial disconnection from nature during the industrialisation of Finland, and a partial reconnection due to current global trends in organic food consumption. Indeed, after WWII, wild food plants became an important source of income in North Karelia as well as a food source for the local population, representing an alternative to the otherwise fairly monotonous diet^43^. As soon as the economy and the standards of living rose in the 1960s and 1970s, knowledge of wild food plants was no longer needed. Growing urbanisation also contributed to the disconnection of people from the surrounding environment and to the consequent erosion of local plant knowledge. Nordic urban settlements are, however, still often embedded in natural environments^32^. This has allowed a shift to a Nordic post-modern society, where berries are a marginal economic activity, but an important resource for recreational activities^44^. The New Nordic Cuisine movement, which began in the 1990s, and the more recent COVID-19 lockdowns have also contributed: we observed a revaluation of, and a reconnection with, wild food plants, in which berry picking is considered a healthy exercise and a useful leisure activity^3, 7, 45^ Another important aspect is the common idea of the “purity” of Finnish products: “Here [in our area] nature is clean. You can collect meadowsweet [*Filipendula ulmaria*] from your own fields, which have not been cultivated for a long time. There are no cars and we have not practised farming” (Karelian woman, born in 1960). The current knowledge about wild food plants in Finnish Karelia seems to be a mixture of a general concept of Nordic lifestyle, remnants of knowledge acquired from past generations, and “new” knowledge acquired through literature, media, the Internet, local food courses, and organic food shops, where wild berries are seen as healthy “superfoods”^40, 46^.

The role of summer cottages (*mökki*) should not be neglected: these mostly wooden houses are immersed in nature, and the increased availability of cars and roads for reaching far into forests have contributed to the fact that many urban inhabitants in Finland have the opportunity to spend weeks and sometimes months in nature, far from any neighbours. This phenomenon is common to several Nordic countries, and also Russian Karelia^47^. Pouta and colleagues^3, 48^ have noted that during vacations it is very likely that people go berry picking with family and friends, spend time at their summer cottage, or simply go for a walk in the forest. Vaara et al.^22^ stated that frequenting summer cottages is an increasing trend in Finland, and it embeds a connectedness between people (particularly children) and the local non-human nature. Despite narratives indicating a decrease in the foraging of wild plants among younger people, official statistics^49^ report that almost 40% of 15- to 24-year-old Finns participated in wild berry picking in 2020.

Finally, we want to highlight three main limitations of this study. First, “Karelians” included not only Karelians who experienced displacement in the 1940s, but also their descendants born in Finland (some of which also had a Finnish parent), while “Finns” included individuals who did not have any Karelian ancestors. Additionally, people classified as (local) “Finns” might have one or two parents who were born outside Finnish North Karelia. Therefore, the terms “Finns” and “Karelians” should be considered as partially arbitrary. Second, most of the interviewees were conveniently selected in Finland, as they were contacted by phone or mail beforehand, and thus our sample might not be entirely representative of the whole population. Interviews in Russia were collected mostly on the spot (i.e. where the plants were found) according to the interviewee’s availability. Third, the interviews were conducted in either Finnish and Russian, which are spoken by the interviewees in the respective countries, rather than in the endangered and increasingly less used Karelian language, and therefore some nuances in meaning might have been lost (although plant names were also collected in Karelian).

Future research should investigate adjacent provinces in Finland and Russia, and also Swedish and Norwegian contexts, to explore similarities and differences, and to eventually identify the contribution of outdoor activities (especially berry picking) in maintaining, and possibly enhancing, LEK and its intrinsic people-nature connection. In Sweden, berry picking has experienced a sharper decline than in Finland, possibly because of the high urbanisation rate (and, therefore, less interaction between urban populations and rural environments), the vanishing practice of preparing wild berry-based foods, and the availability of low-priced wild berries and berry products in markets and grocery stores^50^. We suggest that increasing the opportunities of connecting people, especially (urban) children, with rural environments through foraging, which we observed as a part of the everyday experience of people from a very young age in Finnish Karelia, might represent a basis for developing long-lasting habits of leisure and fitness activities. The example of Finnish Karelia also teaches us to value local wild resources by including them in everyday seasonal gastronomic practices.

## Methods

### Historical background

**Figure 5:**
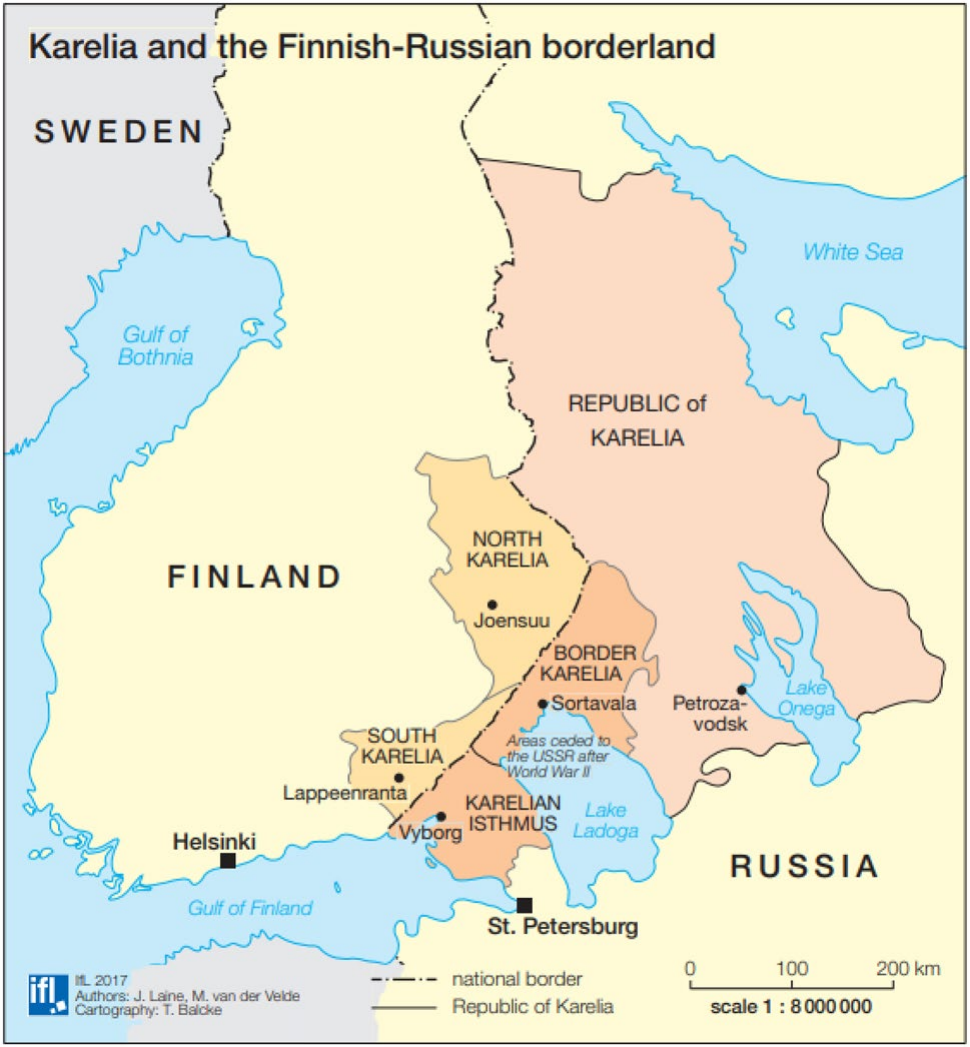
The study area. Reproduced from Laine & Valde^55^ with permission from the first author.

North Karelia is a province in eastern Finland, while the eastern part of Karelia is part of Russia (Soviet Union, beginning in 1944)^51, 52^. North Karelia extends for over 28,000 km^2^ and is inhabited by approximately 162,000 people (Fig. 5). Karelia has been a border area since the 1600s, with cultural and religious influences from both the west (Sweden, Lutheran Christianity) and east (Russia, Orthodox Christianity)^53, 54^.

The situation regarding the displaced Karelians was complicated in Finland for several decades after WWII; as summarised by Loehr et al.^36^: “Karelian evacuees often faced serious prejudice, and many resorted to hiding their Karelian accents and identities to avoid encountering negative reactions”. Many of them were relocated to the Finnish provinces of North and South Karelia^56^. Scott^57^ identifies the following stages in the development of the Karelian population during the past few centuries: Russification (1809–1917), Finnicisation (1917–1939), WWII (1939–1944), and Sovietisation of the Karelians who stayed in the USSR (1945–1991); and we could add Russification during the past two decades. The Karelians in Finland have been intensively Finnicised, and acknowledgement of their ethnic identity and rights has occurred only in the past few decades^58^. The Karelians who remained in the part of Karelia annexed to the Soviet Union were subjected to persecutions, along with many other minorities, in the former USSR^49^, and this situation continues in the present-day Russian Republic of Karelia. The region was partially repopulated with ethnically diverse individuals, who were relocated by the state from other parts of the Soviet Union^49^. Currently 150 ethnicities live in Russian Karelia, mainly Belarusians and Ukrainians^59^.

### Data collection

We conducted 67 semi-structured interviews with 33 Karelians and 34 Finns in Finland during spring and summer 2018. One Karelian and one Finnish interviewee did not name any wild food plants and were thus excluded from the analysis. Interviewees were purposely selected and contacted before the interviews in the province of North Karelia (the municipalities of Joensuu, Lieksa, Liperi, Polvijärvi, and Nurmes) and in Helsinki, starting with personal contacts of the authors and then applying the snowball method. Interviews consisted of questions on wild food plants, including their use and preparation, and periods of use. Interviews were conducted in Finnish, although a few respondents occasionally answered using the Karelian language.

The participants in the study had an average age of 68 years, and comprised 41 women and 26 men with varying degrees of formal education. Nineteen study participants possessed higher education, 29 secondary education, 17 primary education and two had no formal education. The majority of the interviewees lived in rural contexts, while 10 study participants lived in the larger urban areas of Helsinki or Joensuu.

The interviews lasted between 30 min and three hours and were recorded upon permission. The ISE guidelines were strictly followed: prior informant consent was verbally obtained, and and written informed consent was obtained later during or after the interview. The research received approval by the Ethics Committee of the Ca’ Foscari University of Venice. All research methods were performed in accordance with the ISE guidelines and the Declaration of Helsinki.

The recordings were transcribed, and then the ethnobotanical data were extrapolated, plant taxa were identified, and data were entered in an Excel table.

Data collection in Russian Karelia (Petrozavodsk, Republic of Karelia, Russian Federation) took place in the summers of 2018 and 2019. This included semi-structured interviews among 29 Karelians and 21 Russians conveniently selected. Interviews were conducted in Russian by the sixth author (more details in Kolosova et al.^25^).

Dried specimens and plants were collected during the interviews, identified by the eighth author, and later stored at the Herbarium of Ca’ Foscari University of Venice (UVV), bearing accession numbers UVVETBOTKAR01-39 and UVVETBOTKARDR01-31. The collection of fresh and dried specimens with the assistance of study participants was especially important for correctly identifying the species.

### Data analysis

The data was organised into detailed use reports (DUR), which included, among other things, interview code, local name of the plant, the plant’s scientific name and family, part(s) of the plant used, food preparation, and period of use. For instance, each interviewee could report several DURs for the same plant taxon when several foods were prepared (e.g. *Vaccinium myrtillus* could be used by the same interviewee in making *survo* (mashed berries)*, ruispuuro* (rye porridge), and jam, thus resulting in three DUR). The DUR were then analysed, using MS Access and Excel, to produce cross-border and cross-cultural Venn diagrams and to calculate the Jaccard Similarity index. We calculated the Jaccard Similarity index as follows: (C/(A + B − C)) × 100, where C is the number of uses common to A and B, A is the number of uses in sample A, and B is the number of uses of sample B^60^.

## Supplementary Information

Supplementary Information.

## Data Availability

The datasets generated and/or analysed during the current study are available from the corresponding author on reasonable request.
