# Supplementary material for: Outdoor activities foster local plant knowledge in Karelia, NE Europe
Source: Sci Rep. 2023 May 27;13:8627. doi: 10.1038/s41598-023-35918-7 (PMC10220336; doi:10.1038/s41598-023-35918-7)
Supplement: Supplementary file 1 — Supplementary Information. [file 41598_2023_35918_MOESM1_ESM.docx]

**APPENDIX TABLE**

*Table 1: Uses of wild edible plants among Finns and Karelians living in Finnish Karelia; * refers to a past use; AD refers to a recently adopted use; AB refers to a recently abandoned use; T to a temporal use (i.e. a recipe which was adopted and then abandoned). Uses with more than 10 DUR are in bold.*

| **Latin name (Family)**  Local name (Fin – Finnish; Kar – Karelian) | **Food use** | **FINNISH** | **KARELIANS** |
| --- | --- | --- | --- |
| ***Achillea millefolium* L. (Asteraceae)**  Siankärsämö, siankärsmö (Fin) | Tea (aerial parts) | 1* | 2AD |
| ***Aegopodium podagraria* L. (Apiaceae)**  Vuohenputki (Fin) | Salad (aerial parts) |  | 1AD |
| ***Alchemilla* sp. (Rosaceae)**  Poimulehti (Fin) | Salad (aerial parts) |  | 1AD |
| ***Alnus* sp. (Betulaceae)**  Leppä (Fin) | Smoking (wood) | 5 | 9 |
|  | Condiment (wood) | 1* |  |
|  | Bread additive (leaves) | 1T |  |
| ***Angelica archangelica* L. (Apiaceae)**  Väinönputki (Fin) | Salad (young shoots) | 1AD |  |
| ***Arctostaphylos uva-ursi* (L.) Spreng. (Ericaceae)**  Sianpuolukka (Fin); Poččin buola (Kar) | Snack (fruit) |  | 1* |
| ***Betula* spp. (Betulaceae)**  Koivu (Fin) | Tea (leaves) | 4 | 3T |
|  | Snack (leaves and twigs) | 3 | 1AD |
|  | Salad (leaves) |  | 3AD |
|  | Drink (sap) | 9 | 9 |
| ***Calluna vulgaris* (L.) Hull (Ericaceae)**  Kanerva (Fin) | Condiment for pastry (flowers) | 1 |  |
|  | Tea |  | 1 |
| ***Doronicum* sp. (Asteraceae)**  Vuohenjuuri (Fin) | Condiment |  | 1AD |
|  | Pesto |  | 1AD |
| ***Carum carvi* L. (Apiaceae)**  Kumina (Fin) | Condiment | 4 |  |
| ***Centaurea cyanus* L. (Asteraceae)**  Ruiskaunokki (Fin) | Decoration (tea) | 1AD |  |
| ***Chenopodium album* L. (Amaranthaceae)**  Saviheinä (Fin) | Pesto (leaves) | 1AD |  |
|  | Salad (leaves) | 1AD |  |
| ***Elymus repens* (L.) Gould (Poaceae)**  Juolavehnä (Fin) | Snack (aerial parts) | 2* |  |
| ***Empetrum nigrum* L. (Ericaceae)**  Variksenmarja, kaarnikka (Fin) | Juice (fruit) | 1* |  |
|  | Snack (fruit) | 1* |  |
| ***Epilobium angustifolium* L. (Onagraceae)**  Horsma (Fin) | Tea (aerial parts) | 1 |  |
|  | Salad like asparagus (shoots) | 2 | 3 |
|  | Tea (flower buds and leaves) |  | 2 |
|  | Condiment (leaves) |  | 1* |
| ***Hylotelephium telephium* (L.) H. Ohba (Crassulaceae)**  Maksaruoho (Fin) | Condiment (leaves) | 1* |  |
| ***Fragaria vesca* L. (Rosaceae)**  Mansikka, ahomansikka, metsämansikka luonnon mansikka (Fin);meččumandžöi,mandžöi, man’dž’oi, mantsoi, mansoi (Kar) | Decoration (fruit) | 1* | 1 |
|  | Dessert (fruit) | 1AD | 2 |
|  | Jam (fruit) | 1* | 2 |
|  | Kissel [thick fruit soup] (fruit) | 2* | 2 |
|  | Pie (fruit) | 4 | 1* |
|  | Porridge (fruit) | 1 |  |
|  | Preserved (fruit) |  | 1* |
|  | Puree (fruit) |  | 1* |
|  | smoothie |  | 1AD |
|  | **Snack** (fruit) | **22** | **21** |
|  | *Survos* [mashed berries] (fruit) |  | 1* |
|  | With ice cream (fruit) | 3* |  |
|  | With milk (and sugar) (fruit) | 5* | 4* |
|  | With pancakes (fruit) |  | 1AD |
|  | With sugar (fruit) |  | 1 |
|  | With sour milk (fruit) | 1AD |  |
| ***Juniperus communis* L. (Cupressaceae)**  Kataja (Fin) | Condiment (fruit) | 2 | 3* |
|  | For smoking (twigs) | 5 | 7 |
| ***Linnaea borealis* L. (Caprifoliaceae)**  Vanamo (Fin) | Snack (aerial parts) | 1* |  |
| ***Malva* sp. Malvaceae)**  Malva (Fin) | Salad (aerial parts) | 1AD |  |
| ***Oxalis acetosella* L. (Oxalidaceae)** Ketunleipä, käenkaali (Fin); reboinleibü (Kar) | **Snack (leaves)** | **12** | 5 |
| ***Phleum* sp. (Poaceae)**  Timotei (Fin) | Snack (stem) | 1* |  |
| ***Picea abies* (L.) H.Karst. (Pinaceae)**  Kuusi (Fin) | Condiment (shoots) |  | 2* |
|  | Jam (shoots) | 1AD | 1AD |
|  | Jelly (shoots) | 2AD | 1AD |
|  | Snack (shoots) | 5 | 2AD |
|  | Syrup (shoots) | 2 | 3 |
|  | With ice-cream (shoots) | 1AD |  |
|  | Decoration (shoots) | 1* |  |
|  | Drink (shoots) | 1* |  |
| ***Pinus* sp. (Pinaceae)**  Mänty (Fin) | Bread additive (inner bark) | 1* |  |
| ***Polypodium vulgare* L. (Polypodiaceae)**  Kallioimarre (Fin) | Snack (rhizome) | 1* |  |
| ***Populus tremula* L. (Salicaceae)**  Haapa (Fin) | For smoking (wood) | 2 |  |
| ***Ribes nigrum* L. (Grossulariaceae)**  Musta viinimarja, musta herukka (Fin) | Tea (leaves) | 1* | 2 |
|  | Drink (leaves) | 1AD |  |
|  | Preserves (leaves) |  | 1* |
|  | Condiment (leaves) |  | 1* |
| ***Rhododendron tomentosum* Harmaja (Ericaceae)**  Suopursu (Fin) | Tea (aerial parts) |  | 1AD |
| ***Rosa* sp*.* (Rosaceae)**  Ruusu, villiruusu, ruusunmarja (Fin) | Jam (fruit) | 1T | 1 |
|  | Snack (fruit) | 2* | 3* |
|  | Porridge (fruit) |  | 1 |
|  | Tea (aerial parts) | 1 |  |
|  | With yogurt (fruit) |  | 1 |
| ***Rubus arcticus* L. (Rosaceae)**  Mesimarja, mesikka (Fin); orhoi (Kar) | Condiment (fruit) | 1 |  |
|  | Decoration (fruit) | 1* |  |
|  | Jam (fruit) |  | 3* |
|  | Juice (fruit) |  | 1* |
|  | Kissel [thick fruit soup] (fruit) | 1 | 1* |
|  | Porridge (fruit) |  | 1 |
|  | **Snack (fruit)** | **11** | **24** |
|  | Tea (fruit) | 1AB | 6* |
|  | With milk (and sugar) (fruit) | 2* |  |
| ***Rubus chamaemorus* L. (Rosaceae)**  lakka, suomuurain, keltamarja, muurain, suomarja, hilla (Fin); hillo/hilloi, muurain, muuroi - not ripened (Kar) | Candied (fruit) | 1* |  |
|  | Decoration (fruit) | 2* | 1* |
|  | Dessert (soup) (fruit) | 1 | 4 |
|  | **Jam** (fruit) | **18** | **15** |
|  | Jam (with ice-cream) (fruit) | 1 |  |
|  | Juice (fruit) |  | 1 |
|  | Kissel [thick fruit soup] (fruit) | 4 | 9 |
|  | Liqueur (fruit) |  | 1* |
|  | Pastry (fruit) | 1* | 1* |
|  | **Pie** (fruit) | **8** | **20** |
|  | Porridge (fruit) | 3 | 3 |
|  | Preserved (fruit) | 1* |  |
|  | Preserved in alcohol (fruit) | 1 | 1* |
|  | Preserved with sugar (fruit) |  | 1* |
|  | Smoothie (fruit) |  | 1AD |
|  | **Snack** (fruit) | **12** | **7** |
|  | Sorbet (fruit) |  | 1 |
|  | *Survos* [mashed berries] (fruit) |  | 1* |
|  | Tea (fruit) |  | 2* |
|  | With bread (fruit) | 1 |  |
|  | With cheese (fruit) | 1* |  |
|  | With cream (fruit) |  | 1* |
|  | With ice cream (fruit) | 7 | 8 |
|  | With milk (and sugar) (fruit) | 5* | 5* |
|  | With pancake (fruit) | 1 | 1ab |
|  | With sugar (fruit) |  | 3 |
|  | With sour milk (fruit) | 3AD | 3 |
| ***Rubus idaeus* L. (Rosaceae)**  Vattu, vaapukka/ vaabukka, vadelma, vaarain (Fin) vagoi, vabuška, vavoi, vuapoi (Kar) | Candied (fruit) | 1* |  |
|  | Condiment (soup) (fruit) |  | 1* |
|  | Decoration (fruit) | 2 | 3 |
|  | Dessert (soup) (fruit) |  | 2 |
|  | **Jam** (fruit) | **18** | **16** |
|  | Jam (frozen) (fruit) | 1 |  |
|  | Juice (fruit) | 3 | 4 |
|  | Kissel [thick fruit soup] (fruit) | 3 | 7 |
|  | Muesli (fruit) | 1AD |  |
|  | Pastry (fruit) | 1 |  |
|  | **Pie** (fruit) | **12** | **15** |
|  | Porridge (fruit) | 2 | 6 |
|  | Smoothie (fruit) |  | 1AD |
|  | Snack (fruit) | 7 | 9 |
|  | *Survos* [mashed berries] (fruit) | 1 |  |
|  | Tea (fruit) | 8 | 5 |
|  | Wine (fruit) | 1AD |  |
|  | With ice cream (fruit) | 7 | 6 |
|  | With milk (and sugar) (fruit) | 2 |  |
|  | With pancakes (fruit) | 2* | 1* |
|  | With sugar (fruit) |  | 1* |
|  | With sour milk (fruit) | 1 | 3 |
| ***Rubus saxatilis* L. (Rosaceae)**  Lillukka (Fin) | Kissel [thick fruit soup] (fruit) |  | 1* |
|  | Snack (fruit) | 2* | 1* |
| ***Rumex acetosa* L. (Polygonaceae)**  Suolaheinä (Fin); suoluheinü (Kar) | Condiment (aerial parts) |  | 1* |
|  | Snack (aerial parts) | 15 | 12 |
|  | Salad (aerial parts) | 1T |  |
| ***Sorbus aucuparia* L. (Rosaceae)**  Pihlaja (Fin) | Drink (fruit) |  | 1* |
|  | Jam (fruit) | 2 | 1* |
|  | Jelly (fruit) | 1AD | 4 |
|  | Juice (fruit) |  | 2* |
|  | Decoration (fruit) | 1* |  |
|  | Snack (fruit) |  | 1* |
|  | Wine (fruit) | 1AD |  |
|  | *Vispipuuro* [whipped porridge] |  | 1* |
| ***Syringa vulgaris* L. (Oleaceae)**  Syreeni (including: pihasireeni, Unkarin sireeni) (Fin) | Decoration (flower) | 1AD |  |
| ***Taraxacum officinale* F.H.Wigg. s.l. (Asteraceae)**  Voikukka (Fin) | Wine (aerial parts) | 1AD |  |
|  | Colouring (flowers) | 1AD |  |
|  | Kissel [thick fruit soup] (flowers) |  | 1T |
|  | Salad (aerial parts) | 6AD | 4AD |
|  | Drink (roots) | 1* |  |
| ***Trifolium pretense* L. (Fabaceae)**  Puna-apila (Fin) | Tea (aerial parts) | 1AD | 3AD |
| ***Trifolium repens* L. (Fabaceae)**  Valko-apila (Fin) | Tea (flower) | 1AD | 3AD |
| ***Urtica dioica* L. (Urticaceae)**  Nokkonen, viholainen (Fin); čiiloi heinü, šiiloi heinü, vihulainen (Kar) | Condiment (aerial parts) | 2 | 2 |
|  | For baking (aerial parts) | 1 |  |
|  | Pancake (aerial parts) | 3T | 1 |
|  | Pie (aerial parts) | 1AD |  |
|  | Salad (aerial parts) | 2AD | 1AD |
|  | Soup (aerial parts) | 8 | 3 |
|  | Spread (aerial parts) | 1 | 3 |
|  | Tea (aerial parts) | 5 | 4 |
|  | Bread additive (seeds) | 1 | 1AD |
| ***Vaccinium myrtillus* L. (Ericaceae)**  Mustikka (Fin); mušt’oi, mustčöi, muštöi, muštoi, must’oi, mustoi (Kar) | Condiment (fruit) |  | 2* |
|  | Decoration (fruit) | 2 | 1 |
|  | **Dessert (soup)** (fruit) | **9** | **14** |
|  | **Jam** (fruit) | **19** | **18** |
|  | Jam (frozen) (fruit) | 1 | 1 |
|  | **Juice** (fruit) | **13** | **16** |
|  | **Kissel** (fruit) | **11** | **14** |
|  | Pastry (fruit) | 4 | 2* |
|  | **Pie** (fruit) | **24** | **28** |
|  | Pie (in yeast dough) (fruit) | 1* |  |
|  | Pie (*mustikkapiirakka*) (fruit) |  | 1* |
|  | Porridge (fruit) | 7 | 10 |
|  | Puree (fruit) |  | 1 |
|  | Ruispuuro [rye porridge] (fruit) | 1* |  |
|  | Smoothie (fruit) | 1AD | 2AD |
|  | **Snack** (fruit) | **11** | **7** |
|  | Survos [mashed berries] (fruit) |  | 1 |
|  | Tea (fruit) | 7 | 1* |
|  | Wine (fruit) | 1AD |  |
|  | With curd (fruit) |  | 1 |
|  | With ice cream (fruit) | 6 | 8 |
|  | **With milk (and sugar)** (fruit) | **14** | **17** |
|  | With milk and dried oats (fruit) | 1 |  |
|  | With muesli (fruit) | 3 |  |
|  | With pancakes (fruit) |  | 1 |
|  | With sour milk (fruit) |  | 1 |
|  | With sugar (fruit) |  | 2 |
|  | With sour milk (fruit) | 7 | 6 |
| **Vaccinium oxycoccos L. (Ericaceae)**  Karpalo (Fin); garbalo, karpalo, karbalo (Kar) | Candied (fruit) | 1 |  |
|  | Decoration (fruit) | 1 | 1* |
|  | Dessert (fruit) | 1 |  |
|  | Dessert (soup) (fruit) | 1 | 1AB |
|  | Jam (fruit) | 3 | 3 |
|  | Jelly (fruit) |  | 3AD |
|  | Juice (fruit) | 7* | 10 |
|  | Kissel [thick fruit soup] (fruit) | 9* | 8 |
|  | Milkshake (fruit) |  | 1AD |
|  | Pie (fruit) |  | 1* |
|  | Porridge (fruit) | 2 | 4 |
|  | Snack (fruit) | 4 | 7 |
|  | Sorbet (fruit) | 1* |  |
|  | With buttermilk (fruit) | 1* |  |
|  | With cabbage casserole (fruit) | 1* |  |
|  | With ice cream (fruit) |  | 3 |
|  | With sour milk (fruit) | 1 |  |
|  | With muesli (fruit) | 1AD |  |
| ***Vaccinium uliginosum* L. (Ericaceae)**  Juolukka, koiranmarja (Fin) juomoi, juoloi (Kar) | Dessert (soup) (fruit) |  | 1* |
|  | Jam (fruit) | 1 |  |
|  | Snack (fruit) | 1* | 5* |
| ***Vaccinium vitis-idaea* L. (Ericaceae)**  Puolukka, puolain (Fin); buola/ puola, puoloi, buoloi (Kar) | Biscuits (fruit) |  | 1AD |
|  | Bread additive (fruit) | 1* |  |
|  | Dessert (fruit) |  | 1* |
|  | Dessert (soup) (fruit) | 1 | 3 |
|  | Jam (fruit) | 9 | 4 |
|  | Jam (with meat) (fruit) | 1* | 2 |
|  | Jam (with pumpkins) (fruit) | 1* |  |
|  | **Juice** (fruit) | **19** | **20** |
|  | Juice out of jam (fruit) | 1 |  |
|  | Kissel [thick fruit soup] (fruit) | 6* | 12* |
|  | Pastry (fruit) | 2* | 2* |
|  | **Pie** (fruit) | **14** | **17** |
|  | Pie [Karelian pie, big pie] (fruit) | 2* |  |
|  | **Porridge** (fruit) | **12** | **12** |
|  | Preserved (fruit) | 2 |  |
|  | Pudding (fruit) | 1* |  |
|  | Puree (fruit) | 1 | 2 |
|  | ***Ruispuuro* [rye porridge]** (fruit) | **11** | **15** |
|  | Smoothie (fruit) | 2AD | 1AD |
|  | Snack (fruit) | 7 | 8 |
|  | Sorbet (fruit) | 1* |  |
|  | *Survos* [mashed berries] (fruit) | 6 | 10 |
|  | Tea (fruit) | 2T |  |
|  | ***Vispipuuro* [whipped porridge]** (fruit) | **12** | **18** |
|  | Wine (fruit) | 1AD |  |
|  | With blood pancake (fruit) | 2 |  |
|  | With curd (fruit) |  | 1AD |
|  | With ice cream (fruit) | 3 | 10 |
|  | With milk (and beestings/sugar/talkkuna [mix of flours]) (fruit) | 5 | 10 |
|  | With muesli (fruit) | 2AD |  |
|  | With oatmeal (fruit) | 1* |  |
|  | With pancakes (fruit) | 1 |  |
|  | With stuffed cabbage (fruit) | 1 |  |
|  | With sugar (fruit) | 2* | 2 |
|  | With sour milk (fruit) | 5AD | 4 |
| ***Valeriana officinalis* L. (Caprifoliaceae)**  Rohtovirmanjuuri, valeria (Fin) | Condiment (N.D.) |  | 1AD |
|  | Tea (N.D.) |  | 1AD |
| ***Vicia cracca* L. (Fabaceae)**  Hiirenvirna (Fin) | Salad (shoots) | 1AD |  |
